# Group singing is globally dominant and associated with social context

**DOI:** 10.1098/rsos.230562

**Published:** 2023-09-06

**Authors:** Dor Shilton, Sam Passmore, Patrick E. Savage

**Affiliations:** ^1^ Cohn Institute for the History and Philosophy of Science and Ideas, Tel Aviv University, Tel Aviv, Israel; ^2^ Evolution of Cultural Diversity Initiative, Australian National University, Canberra, Australia; ^3^ Graduate School of Media and Governance, Keio University, Fujisawa, Japan; ^4^ School of Psychology, University of Auckland, Auckland, New Zealand; ^5^ Faculty of Environment and Information Studies, Keio University, Fujisawa, Japan

**Keywords:** music, cross-cultural, cultural evolution, social organization

## Abstract

Music is an interactive technology associated with religious and communal activities and was suggested to have evolved as a participatory activity supporting social bonding. In post-industrial societies, however, music's communal role was eclipsed by its relatively passive consumption by audiences disconnected from performers. It was suggested that as societies became larger and more differentiated, music became less participatory and more focused on solo singing. Here, we consider the prevalence of group singing and its relationship to social organization through the analysis of two global song corpora: 5776 coded audio recordings from 1024 societies, and 4709 coded ethnographic texts from 60 societies. In both corpora, we find that group singing is more common than solo singing, and that it is more likely in some social contexts (e.g. religious rituals, dance) than in others (e.g. healing, infant care). In contrast, relationships between group singing and social structure (community size or social differentiation) were not consistent within or between corpora. While we cannot exclude the possibility of sampling bias leading to systematic under-sampling of solo singing, our results from two large global corpora of different data types provide support for the interactive nature of music and its complex relationship with sociality.

## 1. Introduction

Music is an important interactive technology to form, strengthen, and maintain affiliative relationships [1–3]. It leverages the positive effect of behavioural alignment on attachment formation by making interactions more synchronized; specifically, by shaping vocalizations so that they can be more easily imitated, and through the elicitation of a beat to which participants can entrain [3–7]. Emphasizing the importance of interaction in music challenges the notion that it is primarily made for different forms of consumption, and instead suggests that many of its features evolved to encourage simultaneous participation in affiliative interactions involving multiple participants [6,8]. This function is supported by the fact that across cultures, music making is often a participatory activity involved in important religious rituals and communal events [9–11].

However, the way people engage with music in different cultures is not homogenous. The frequency, context, and dynamics of group performance in different societies has the potential to tell us about how these cultures construct social roles and relationships and, eventually, create social order [12]. In post-industrial societies, the interactive and communal function of music is diminished, and music is often more ‘presentational’ than ‘participatory’ [13]. In live performances audiences and musicians are usually separated, and people increasingly listen to pre-recorded music by themselves [13,14]. It is also common for hierarchies to exist between musicians, with soloists or conductors dominating the performance. This is in contrast to participatory music making, where everyone present can participate in the singing, playing and dancing, and which prioritizes communal well-being through broad engagement in the performance over the aesthetic qualities of the sound produced. This suggests that large, hierarchical societies tend toward presentational performance and solo singing, whereas small or more egalitarian societies emphasize participation and group singing [15]. There are at least four non-mutually exclusive hypotheses for this relationship. First, that musical structure reflects social structure, so that in egalitarian societies, singing will be more group-oriented and less differentiated, whereas in hierarchical societies, participants will organize more strictly into roles, with some dominating the performance [12,15]. A notable example is the Central African BaYaka, whose way of singing and dancing together reflects and reproduces their egalitarian ethos by encouraging the participation and varied contribution of all group members, without elevating one voice over the others [16]. A second hypothesis, somewhat related to the first, is that elite strata (e.g. royal courts, nobility) accommodate the emergence of professional musicians for displays of social and aesthetic superiority, establishing performance norms in which musicians and audience are clearly distinguished, and which eventually radiate outside elite circles [17,18]. Alternatively, less participatory forms of music could have been the result of changing demographics, so that as local community sizes increase, coordinated participation becomes more difficult and required specialists to lead the performance [3]. Finally, if musical structure is determined primarily by its social function [10], the typical contexts in which music is performed in a culture will determine whether it leans more toward solo or group singing.

The current evidence for the prevalence of group singing and its relationship to social organization remains limited. Savage *et al*. [19] found group performance to be more common than solo in coded recordings from the Garland Encyclopedia of World Music; however, their sample was relatively small (304 audio recordings, including a substantial portion of instrumental recordings without singing), and their study did not look into relationships with social structure. There have been two other major attempts to quantify global music diversity and its relationship to social function and organization: the Global Jukebox (GJB) [20] and the Natural History of Song (NHS) [10]. The GJB team collected 5776 song recordings from 1024 cultures and coded them according to the social organization of performers and other musical style variables [12,20]. This corpus was recently digitized and made public, and some of Lomax's original findings relating song structure to social complexity have been replicated by more robust analyses; specifically, song structure features that are associated with solo singing (monophony, clear articulation, ornamentation) were significantly predicted by factors related to social complexity [20]. The NHS was created to describe musical diversity and its relationship to social function [10], and consists of 4709 coded ethnographic texts (NHS Ethnography) describing song performance in a globally representative and historically independent sample of 60 societies, along with 118 audio recordings of songs from 86 societies (NHS Discography). By combining the GJB and NHS Ethnography corpora—a combined sample of over 8600 recordings and texts—we test the prevalence of group singing and the putative correlates of change in participatory dynamics related to social organization.

## 2. Results

### 2.1. Group singing is globally dominant and varies between geographical regions

**Figure 1.**
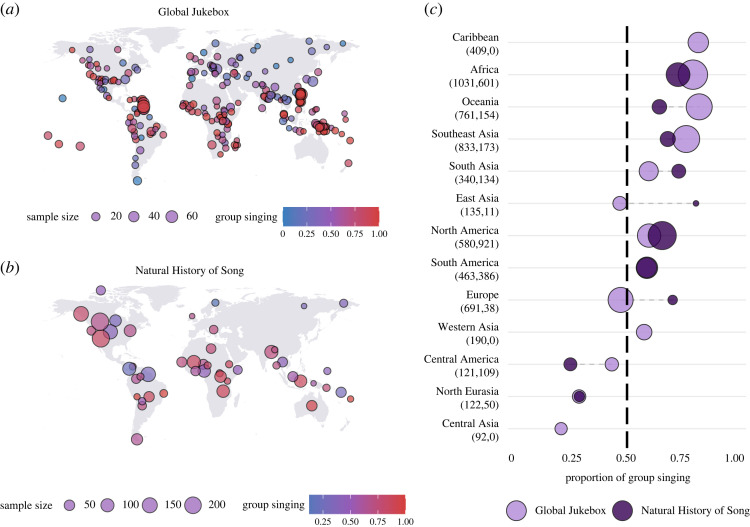
Global and regional prevalence of group singing. (*a*) Location of 247 societies with at least 10 recordings in the Global Jukebox (GJB). Societies with the same Ethnographic Atlas ID are merged. (*b*) Location of 49 societies with at least 10 texts in the Natural History of Song Ethnography (NHS). (*c*) Proportion of group singing in every geographical region. Circles are sized based on regional sample sizes, displayed in parentheses (number of GJB songs, number of NHS texts).

Group singing—defined as simultaneous singing of more than one person—is predominant in both corpora (figure 1). It accounts for 67% of all songs in GJB (3883 out of 5776 songs) and 65% of all coded ethnographic texts in NHS (1676 out of 2581 texts without missing data). Of societies with at least 10 songs/texts (GJB: *n* = 240 societies; NHS: *n* = 49), 70% in GJB and 73% in NHS have more group than solo singing, and the average of group songs per society is 66% in GJB and in NHS. While most societies contain a combination of solo and group singing performances, there are more societies in which solo singing is absent than vice versa: among societies with at least 10 entries, 22% have no solo singing in GJB (compared to 4% with no group singing), and 6% have no solo singing in NHS (compared to none with no group singing). Societies that were present in both the GJB and NHS (*n* = 42) show comparable levels of group singing: a median difference of 24%, significantly lower than chance (permutation *z* = −2.05, *p* = 0.01). When considering societies with at least 10 entries (*n* = 17), the difference is 15% (see electronic supplementary material for methodological details). Among the various forms of group singing in GJB (a level of detail not available in NHS), the most common are antiphonal call and response between a soloist and a group (31%, *n* = 1940, codes 8, 10, 11), followed by unison singing (21%, *n* = 1351, codes 5, 6). The least common is diffuse singing (3%, *n* = 164, code 7), in which every participant sings independently and in minimal coordination with others (see electronic supplementary material, figure S1). We consider possible sampling biases such as the under-sampling of solo singing in the Discussion section.

There are notable differences in the prevalence of group singing between geographical regions. Group singing is clearly predominant in Africa, Oceania and Southeast Asia, while solo singing is predominant in North Eurasia and Central America (figure 1*c*). Estimates of regional group singing dominance are similar between datasets, with two exceptions: Europe and East Asia, which differ by 24% and 35% between GJB and NHS. However, Europe and East Asia also have the smallest society and text samples in NHS (two societies each, regional text samples of 38 and 11 texts respectively, compared with a median regional text sample of 144). Furthermore, unlike GJB, NHS Ethnography does not include many large-scale ethnolinguistic groups (e.g. Japanese, Han Chinese, English, Arab), societies that are common in Eurasia and where solo singing tends to be predominant. GJB also has data for two regions that are not represented in NHS: the group-predominant Caribbean (82% group singing, song sample = 409) and solo-predominant Central Asia (17% group singing, song sample = 92).

### 2.2. Group singing is associated with social context

**Figure 2.**
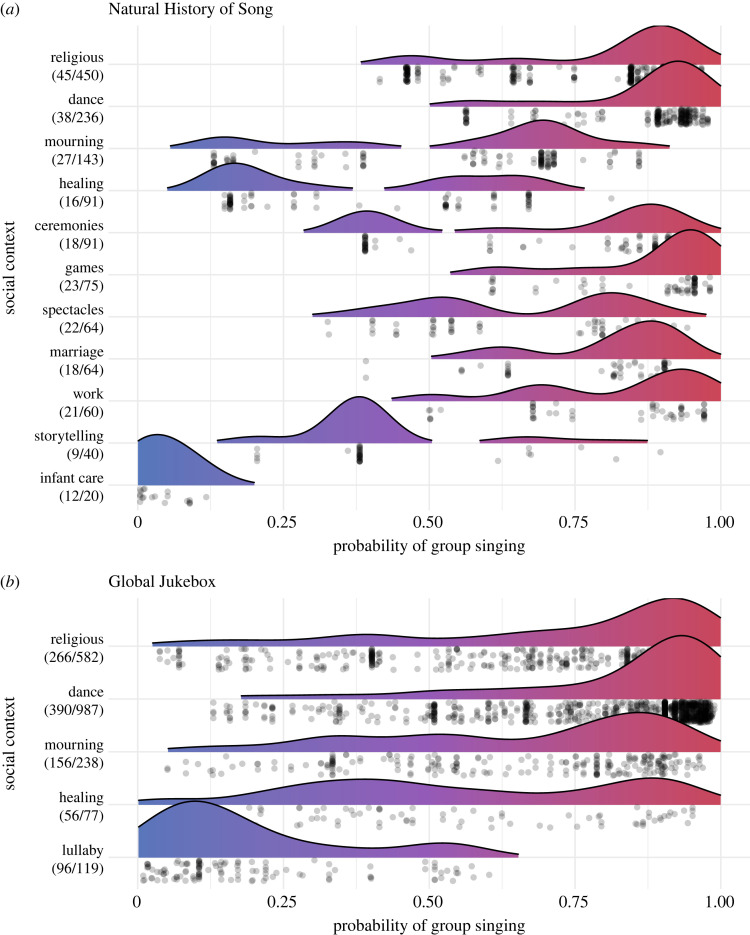
Relationship between group singing and social context. Probability of group singing for each text/recording as predicted by social context and society random effects, for Natural History of Song (*a*) and Global Jukebox (*b*). For each context, the number of societies and number of texts are listed in parentheses. Datapoints shown below ridge.

Before considering social organization, we estimate the correlation of group singing with social context. In NHS, 62 primary contexts were coded, most of which appear only a few times. We therefore limit our analysis to 11 social contexts that are the most common and whose association with singing is best supported [9], resulting in a subset of 1334 texts from 58 societies. The social contexts for which group singing proportion is highest are religious practices (0.8, *n* = 450), dance (0.87, *n* = 236), games (0.88, *n* = 75), marriage (0.8, *n* = 64) and work (0.82, *n* = 60). The most solo-dominant contexts are infant care (0.05, *n* = 20), healing (0.38, *n* = 91), storytelling (0.42, *n* = 40) and mourning (0.55, *n* = 143). To consider the strength of social context as a predictor, we fit two binomial multilevel models with group/solo singing as the response: a model with random intercepts for societies and no fixed effect, and a model with social context as a predictor in addition to the random society intercepts. We find that the social context model is a significantly better fit and explains a substantial amount of variation (ΔAIC = 103, conditional *R*^2^ = 0.41, marginal *R*^2^ = 0.16; figure 2*a*).

Since social context was not coded in GJB, we tested an automatic procedure based on metadata keyword search for five primary contexts: religious practices, dance, mourning, healing and lullabies (corresponding to infant care in NHS). Using a random sample of 200 songs, we found automatic and manual coding have a high inter-rater reliability (Cohen's Kappa: religious = 0.64, dance = 0.91, mourning = 0.49, healing = 1, lullabies = 1). Running this automated procedure on the entire corpus produced a subset of 1773 songs from 598 societies for which at least one social context was coded. Group singing proportions for each context are in the same rank order as NHS but spread across a smaller range of values, as follows: dance (0.8, *n* = 987), religious practices (0.71, *n* = 582), mourning (0.64, *n* = 238), healing (0.56, *n* = 77) and lullabies (0.24, *n* = 119). The GJB context model was likewise preferable to an intercept-only model, explaining a similar proportion of variance (ΔAIC = 152, conditional *R*^2^ = 0.58, marginal *R*^2^ = 0.12; figure 2*b*). These consistent results across corpora suggest social context is an important factor in the likelihood of group singing.

### 2.3. Community size is associated with group singing only in the Global Jukebox

**Figure 3.**
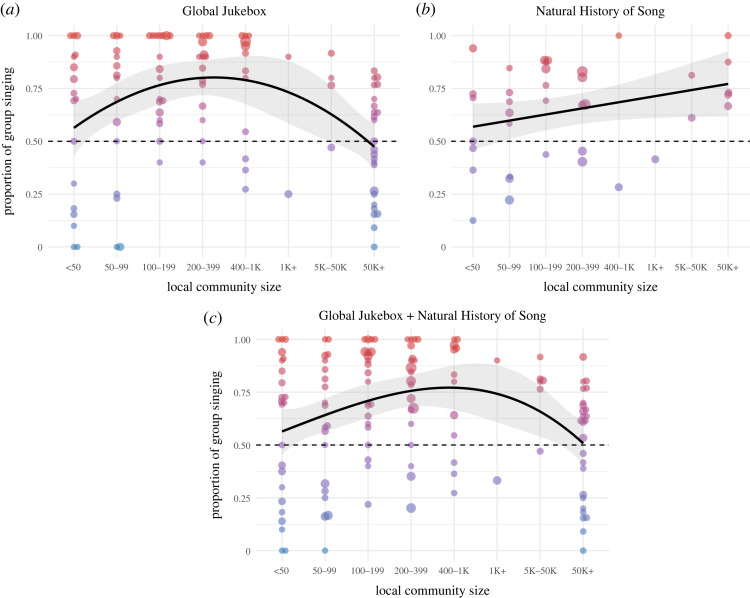
Relationship between group singing and community size. Proportion of group singing and local community size in societies with a sample of at least 10 entries in GJB (*a*), NHS (*b*) and both (*c*). Circle size weighted by number of entries; colour gradient indicates proportion of group singing; shaded areas indicate 95% CI.

Hypotheses about the relationship between group or solo dominance and social organization have focused on community size and social differentiation. To examine the former, we fit binomial multilevel models with group/solo singing as the response, random society intercepts, and the mean size of local communities as a predictor. The mean size of local communities is an ordinal variable coded in the Ethnographic Atlas (EA031), ranging from local communities of under 50 to urban centres of over 50 000 people [21,22]. We find a significant quadratic relationship in GJB (*n* societies = 109, *b* = −8.6, *p* < 0.001, conditional *R*^2^ = 0.46, marginal *R*^2^ = 0.09; figure 3*a*), but no significant relationship (linear or quadratic) in NHS (conditional *R*^2^ = 0.25, marginal *R*^2^ = 0.01; figure 3*b*). We suggest this may be due to the underrepresentation of large-scale societies—especially from Europe and East Asia—in NHS. When fitting a model with both corpora, the quadratic relationship is also significant (*n* societies = 131, *b* = −5.8, *p* < 0.001, conditional *R*^2^ = 0.36, marginal *R*^2^ = 0.04; figure 3*c*). To mitigate the possible influence of autocorrelation, we fit another model including only societies from the stratified Standard Cross-Cultural Sample (SCCS), and find that the quadratic relationship is still significant (*n* societies = 65, *b* = −6.5, *p* = 0.005, conditional *R*^2^ = 0.38, marginal *R*^2^ = 0.05). These results, while somewhat supportive of a relationship between group singing and community size, also indicate this relationship is more sensitive to sampling differences than the relationship to social context.

### 2.4. Social differentiation is not consistently associated with group singing

**Figure 4.**
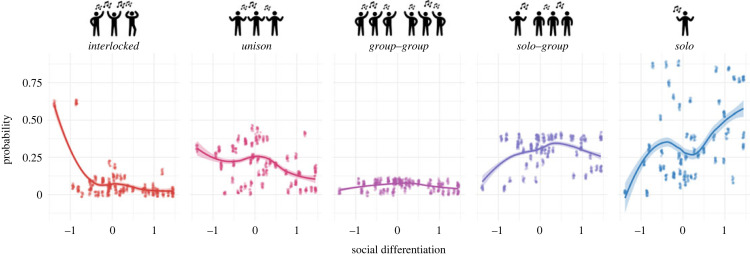
Relationship between sociovocal styles and social differentiation. Each facet shows the probabilities of songs belonging to the specified type of singing as predicted by social differentiation and random society effects. Data from societies with at least 10 songs are shown (641 songs, 50 societies). Left-hand panel shows the skewed predictions for the interlocked response category, attributable to just two societies. Points are jittered (0.02).

Lastly, we test the relationship with social differentiation, i.e. the extent to which a society is segmented into different roles and classes, leading to discrepancies in wealth, power and prestige. We use a latent variable derived from SCCS variables which combines the following measures (coded as ordinal variables): levels of political organization, status distinctions and stratification, use of money, use of written records and technological specialization [23–25]. Societies low on this scale include modern hunter–gatherers such as the African Mbuti and Hadza, both well known for their egalitarian organization [26]. Societies high on this scale include the Japanese [27]. We test whether social differentiation can predict group singing (using the same binomial multilevel models described in previous sections) or, more specifically, the social differentiation of vocalists. To test the latter, we reorganize the original GJB variable describing the social organization of singers into a sociovocal scale ranging from the most integrated group dynamic to the clearest emphasis on a soloist, in the following order: (1) interlocked singing (participants sing distinct yet complementary parts); (2) social unison (participants sing the same melody and text throughout); (3) call and response between two or more groups; (4) call and response between soloist and group; and (5) solo or alternating soloists. We fit Bayesian multilevel cumulative link models with the sociovocal scale as the response, social differentiation as predictor, and random intercepts for societies. We find, first, that social differentiation is an insignificant predictor of group singing (GJB: *b* = −0.73, *p* = 0.07; NHS: *b* = −0.14, p = 0.7). Second, while social differentiation does correlate well with our sociovocal scale (OR = 2.18, 95% CI 1.05–4.44), we find that the effect is driven mostly by the interlocked/unison category, and more specifically, by two societies in which interlocked singing is most dominant: the Ju'hoansi and the Mbuti (figure 4). This means that the positive relationship between social differentiation and the sociovocal scale is driven primarily by the prevalence of interlocked singing among two specific societies with low social differentiation. Notably, these two societies are the same ones which Lomax originally featured in his hypothesis that song structure reflects social structure [15], since in both there are striking parallels between how society is organized and how singers sing together. These results indicate this is not, as Lomax had once suggested, a general relationship operating in many cultural settings, but a regionally specific property of some African hunter–gatherers.

## 3. Discussion

Our findings provide the widest support so far that music-making is predominantly a participatory group activity relying on tonal and rhythmic coordination. Using two different datasets consisting of audio recordings and ethnographic texts, we find compelling converging evidence of the global predominance of group singing, its regional variation, and its relationship to social context. More specifically, in both corpora group singing is more common than solo singing in 70–73% of societies, varies similarly between regions, and is more likely during religious rituals, dancing, playing games, and working, while solo singing is more likely in the context of infant care, healing and storytelling. On the other hand, support for a relationship with community size relies only on GJB data. Specifically, our results indicate that group singing is more common in medium-sized communities of several hundred while solo singing is more common in urban centres of over 50 000. We find no consistent relationship between social differentiation and group singing or the sociovocal differentiation of singers.

The global predominance of group singing is consistent with the ‘music as social bonding’ hypothesis [3], though it may be consistent with other hypotheses. For example, an alternative theory suggesting that music evolved in part to signal social cohesion to other groups also emphasizes music as a coordinated group activity [28,29]. The measurements analysed here are insufficient on their own to distinguish between these theories; instead, one needs to assess the plausibility of each within a wider lineage explanation of human social evolution, itself combining archaeological, psychological, ethnographic and genetic data within an extended evolutionary framework [30–33].

Our analysis showing different relationships with community size in GJB and NHS, which we suggest results from the inclusion or exclusion of larger-scale societies in these corpora, illustrates how using criteria such as sample size, society size, or missing data can inadvertently introduce sampling biases—issues that cross-cultural researchers have been struggling with for generations and continue to struggle with today [34,35]. Another possible sampling issue in both corpora is the under-sampling of certain social contexts simply because field researchers find them less interesting or harder to access. Most notably, NHS Ethnography contains only 20 texts related to infant care, as opposed to 450 related to religious practices, a ratio which we can justifiably assume does not reflect actual frequencies.

More generally, solo singing might be under-sampled in both corpora [36]—particularly when recordings or texts represent social activity witnessed in real time—because it more often occurs in circumstances that are more intimate and therefore less compelling or less accessible to ethnographers. For example, historically ethnographers have tended to be predominantly male, while lullabies have tended to be sung predominantly by women, often in intimate private contexts such as nursing and sleeping in bedrooms, where access may be restricted to outside men. We therefore qualify our analysis as focusing on the more public manifestations of singing, noting also that most social contexts associated with music are indeed public by nature.

Another limitation shared by both GJB and NHS is their focus on vocal performance, which leaves out instrumental forms of group performance and the instrumental accompaniment of soloists. A recent global comparison of song, speech, and instrumental music [37] demonstrates an alternative approach by which both vocal and instrumental recordings can be sampled in a balanced way that includes researchers from each society as coauthors. Yet this approach comes with its own limitations—crucially, all recordings were done solo, making it not a useful comparative resource for comparing rates of solo versus group music-making.

Crucially, both GJB and NHS—and indeed our analysis of these data—are limited by decontextualization and by the relative lack of involvement of researchers from each society in the process of analysing and interpreting their data [34]. As anthropologists and ethnomusicologists have long noted, every social event is suffused with meaning, the interpretation of which requires ‘thick description’ [38]. Thick description can be crucial for decoding the social significance—including the relationship to social organization—of different social behaviours. Here, we only look at one specific dimension of musical practice, noting that there may be others which bear relation to social organization, and that there are multiple factors influencing variation in group singing predominance.

Previous analyses of cantometric data were criticized on several accounts, including the possibility of determining typical song styles for cultures, coding reliability, small sample sizes, and autocorrelation [35,39]. These concerns are substantially mitigated by our research focus and methods. First, the variable we study—the predominant type of singing—has been described by ethnomusicologists for many cultures and even cultural regions, and is a major focus of several ethnomusicological theories (e.g. Turino's distinction between ‘participatory’ and ‘presentational’ music [13]). Seeger [40], for example, explicitly described the two main modes of song among the Suya as solo ‘shout’ songs and unison singing (the GJB sample, provided by Seeger, equally represents both). Interlocked singing is famously the most prevalent form among Mbuti and BaYaka [16,41], as is a different type of interlocked singing among the Kaluli [39]; solo singing has been described as the predominant form in Siberia [42]; and monophonic singing was described as the typical form of group singing across all indigenous North American societies [43]. Since such generalizations are prevalent in the ethnomusicological literature, it seems reasonable that a curated sample could give a good estimate of the typical type of singing in a culture. The corresponding variable in GJB (line 4, ‘Musical organization of the vocal part’) also has the highest inter-rater reliability of all cantometric variables, with Cohen Kappa's scores of > 0.9 and percent agreement values of >80% [20]. The problem of small sample sizes is mitigated by using sample size cutoffs as well as multilevel models which retain within-society variation. The influence of autocorrelation due to historical relationships is somewhat mitigated by using a stratified sample (the SCCS), originally designed to avoid this problem [24]. More importantly, recent work suggests musical styles are weakly correlated with genetic, linguistic and spatial relationships [44]. We therefore have good reasons to consider GJB data as suitable for this kind of study, notwithstanding the inherent tradeoffs one must take into account between sample size and quality. Nevertheless, since social context was shown to significantly affect at least one major feature of song style, we suggest manual coding of this variable in GJB would improve the potential of this corpus for further research.

Though here we focus mainly on social context and organization, future work can help disentangle and clarify the importance of other factors on group singing prevalence. For example, the clear predominance of solo singing in North Eurasia appears to be unrelated to social organization, and the distinctive climate of the region suggests environmental factors may be important, possibly by demanding long periods of relative isolation and thus limiting the frequency of group gatherings. Our models also show the importance of social context. Since religious rituals—one of the most common social contexts—were shown to be strongly associated with group singing, it seems reasonable that in cultures where traditional religious practices were undermined due to missionary and colonial encroachment—as is the case, for example, in several South American indigenous cultures—there would be less group singing overall [36,45,46]. Broadly speaking, the extent to which music is used interactively and for social bonding purposes appears to be related to the type of collective existence humans lead and the ways in which they cooperate [6,12,36]. All factors considered here—social context, community size, social differentiation—relate in some way or another to the frequency and character of group gatherings and collective action, emphasizing once again the main theme of our study: the intimate relationship between music and social life.

## 4. Material and methods

### 4.1. Group singing estimates

The first variable in GJB (line 1) records the social organization of the vocalists on a scale of 1–13 (excluding 3). The codes and their distribution are shown in electronic supplementary material, figure S1. We code all songs with code 2 or 4 as solo, and everything else as group. Code 1 indicates no vocals, and the one recording coded as such (id = 9950) was removed. Because some songs change their sociovocal organization midway, many songs are coded multiple times: 526 songs are coded twice, 25 are coded three times and 2 are coded four times. There are 134 songs which are coded as both solo and group singing, out of which 122 have solo code 4, meaning alternating soloists. When calculating the proportion of group singing, we consider just one code per song. If it is coded both as solo and group, we count it as a group song (coding them as solo changes group singing predominance results by no more than 2%). We include duplicate codes in the cumulative link multilevel models, since these take into consideration the type of group singing. There are also 464 songs with a soloist accompanied by more than one instrument (out of 2027 songs with soloist or alternating soloists). Although these can also be considered as group performances, we found that coding them as ‘group’ did not change our results.

Of the 4709 texts in NHS, 661 texts are duplicates, and 1746 texts (including duplicates) have no information about the number of singers. After removing both, we are left with 2851 texts. In almost all instances, the exact number of singers could not be determined. We recode all values besides ‘Solo singer’ as group singing (see codes and their distribution in electronic supplementary material, figure S2).

### 4.2. Social context analysis

Social contexts in NHS were selected from a curated list of 85 OCM identifiers (ethnography subjects classified in HRAF). Our choice of social contexts for the analysis was based on the 20 most common contexts which also feature among the 20 social contexts described by [10] as universally associated with singing. We excluded one category which did not concretely refer to social interaction (drives and emotions).

Social context in GJB was coded by searching for the following matching texts in the song metadata (name, genre, notes): *Religious*: religious, ritual, rite, pray, initiation, spirit, shaman; *Dance*: dance, dancing; *Mourning*: mourn, lament, funera; *Healing*: heal, cure, curing; *Lullaby*: lullab.

### 4.3. Statistical analysis

All analyses were performed using R v4.2 [47]. Linear and binomial multilevel models were implemented with lme4 v1.1.29 [48], latent variable models with lavaan v0.6–11 [49], and cumulative link multilevel models, due to inadequate performance of frequentist packages, were implemented with brms v2.17.0 [50]. Bayesian models were fitted on four chains with 5000 iterations (1500 warmup), and chain convergence was verified using R-hat and diagnostic plots (trace plots and rank histograms).

## Data Availability

Data and relevant code for this research work are stored in GitHub: https://github.com/dorshilton/group-singing and have been archived within the Zenodo repository [51]: https://doi.org/10.5281/zenodo.8249585. Analysis code is available at https://github.com/dorshilton/group-singing. GJB data are available at https://github.com/theglobaljukebox/cantometrics. NHS data are available at https://github.com/themusiclab/nhs. The data are provided in electronic supplementary material [52].
